# CD27 and GZMK as Co-Biomarkers in Rheumatoid Arthritis and Crohn's Disease and Mediate Immune Imbalance

**DOI:** 10.2174/0118715303372365250421114853

**Published:** 2025-04-29

**Authors:** Li Zheng, Yong Zeng, Miao Fang, Hongquan Heng, Ying Luo, Jie Zhong

**Affiliations:** 1 Department of Orthopedics, Chengdu Second People’s Hospital, Chengdu, China;; 2 Department of Clinical Medicine, Chengdu Medical College, Chengdu, China;; 3 Center for Joint Surgery, The First Affiliated Hospital of Army Medical University, Chongqing, China

**Keywords:** Rheumatoid arthritis, Crohn’s disease, machine learning, WGCNA, immune cell infiltration, immune imbalance

## Abstract

**Background:**

Studies have found similar immune responses, genetic susceptibility, and inflammatory mediators between Rheumatoid arthritis (RA) and Crohn’s disease (CD), but these findings are controversial.

**Methods:**

The Gene Expression Omnibus (GEO) was utilized to get microarray data. Differentially expressed genes (DEGs) in individuals with CD and RA were identified. Weighted gene co-expression network analysis (WGCNA) was used to identify key modular genes in CD and RA. The least absolute shrinkage and selection operator (LASSO) logistic regression was used to identify hub genes. The correlation between immune cell infiltration and common biomarkers was examined by utilizing the GSEA and ssGSEA.

**Results:**

CD27 and GZMK were recognized as co-biomarkers in RA and CD. The analysis of immune infiltration revealed a significant relationship between a range of immune cells, including CD8 T cell, MDSC, and natural killer T cell, and both RA and CD.

**Conclusion:**

CD27 and GZMK are biomarkers shared by CD and RA and are potential diagnostic and therapeutic targets for them, especially in patients with CD combined with RA. CD and RA are highly associated with dysregulation of the acquired immune response system and imbalance of the innate immune system.

## 1 INTRODUCTION

Rheumatoid arthritis (RA) is a persistent inflammatory autoimmune disorder that predominantly impacts the joints. It is distinguished by a symmetrical and progressive inflammation of the joints, which ultimately results in articular cartilage degeneration, bone erosion, disability, and the infliction of pain and inconvenience on over 40 million individuals globally [1, 2]. While RA may not immediately endanger patients’ lives, it can lead to a range of consequences that significantly impact their quality of life and mortality. Worst of all, there is no accurate conclusion on the pathogenesis of RA [3]. Despite extensive research, the precise antigenic triggers and pathogenesis of RA remain poorly understood, particularly in terms of its autoimmune etiology. Crohn’s disease (CD) is a multifactorial inflammatory bowel disease characterized by transmural inflammation and discontinuous “skip lesions” along the gastrointestinal tract [4]. It is one of the most prevalent forms of inflammatory bowel disease, affecting a significant global population. CD can involve any part of the gastrointestinal tract, from the mouth to the rectum, and recurrent inflammation can lead to severe complications, including strictures, fistulas, and life-threatening lesions [3, 5]. Unlike RA, the etiology of CD is partially understood, with strong evidence implicating Mycobacterium avium subspecies paratuberculosis (MAP) as a potential causative agent, and its pathogenesis has been increasingly delineated in recent studies [6].

The pathological process of RA is characterized by three stages: synovial cell proliferation, fibrin exudation, and inflammatory infiltration. The infiltration of proliferative synovial fibroblasts, neutrophils, and monocytes into the synovial tissue drives inflammation, which is central to RA pathogenesis [7]. In contrast, CD is primarily driven by dysregulated immune responses in the intestinal mucosa, with contributions from genetic susceptibility and environmental factors [8]. Despite their distinct etiologies, RA and CD share several common features, including overlapping immune response mechanisms (*e.g*., TNF-α and IL-6 signaling), shared genetic risk factors (*e.g.*, HLA alleles), and similar therapeutic targets (*e.g*., TNF inhibitors) [9]. Epidemiological studies have further highlighted a potential link between these diseases. For instance, a meta-analysis by Chen *et al.* demonstrated an elevated risk of RA in patients with CD [10], while Mendelian randomization analysis by Meisinger *et al.* identified RA as a risk factor for CD [11]. However, the precise mechanisms underlying this comorbidity remain elusive, particularly in terms of shared molecular pathways and immune dysregulation.

Given the shared immune and inflammatory mechanisms between RA and CD, we hypothesized that these diseases may exhibit common molecular pathways and therapeutic targets. To test this hypothesis, we employed an integrative approach combining machine learning, weighted gene co-expression network analysis (WGCNA), and bioinformatics to identify shared genetic signatures and potential diagnostic biomarkers. Functional enrichment analysis (GO and KEGG) and single-sample gene set enrichment analysis (ssGSEA) were further utilized to explore the immune responses and biological processes associated with these shared genes. Our study aims to uncover the molecular basis of RA and CD comorbidity, with a focus on identifying common therapeutic targets and elucidating the immune mechanisms underlying their shared pathogenesis. These findings may provide new insights into the treatment of RA and CD, particularly in cases where these diseases coexist.

## 2 MATERIALS AND METHODS

### 2.1 Data Collection

The gene expression profiles for GSE95095 and GSE1919 were sourced from GEO database(https://www.ncbi.nlm.nih.gov/geo/), which is a comprehensive microarray. The two datasets were based on the GPL14951 platform and the GPL91 platform. The GSE95095 dataset includes intestinal mucosa samples from 24 CD patients and 12 control group patients. The GSE1919 dataset comprises 5 samples obtained from RA patients suffering from synovial membrane and 5 samples obtained from healthy individuals serving as normal participants.

### 2.2 Data Manipulation and Display

We searched for key genes between CD and RA samples using the “limma” package [12]. DEGs were considered noteworthy if their adjusted *p*-value were adjusted *p*-value <0.05 and |log2FC| >1. The heatmap and volcanic graph were then produced by using the “ggplot2” and “pheatmap” R tools [13].

### 2.3 Dentified Key Model Genes by WGCNA

By using the “WGCNA” R package [14], two weighted gene co-expression nets were generated based on the two expression matrices, and common genes linked with both CD and RA were identified. When the samples were first combined, a trustworthy network was created. After that, we determined the Pearson correlation coefficients for every gene pair to evaluate the similarity of expression and produce a correlation matrix. To create a weighted neighborhood matrix, we also used the soft threshold technique. We used a soft interconnection method to find the ideal soft threshold that guarantees scale-free gene interactions. One obtains a topological overlap matrix (TOM) from the neighborhood matrix. Using dynamic tree cutting and guaranteeing at least 100 genes per module, co-expression modules were produced. The calculation of gene significance (GS) and module membership (MM) was performed to establish a connection between modules and clinical features. Ultimately, we successfully plotted eigengenes. Based on the calculation results, the “ggplot2” and “VennDiagram” R package [15] were used to screen the genes with overlapped modules and DEGs. We used the same method to screen for hub genes common to CD and RA.

### 2.4 Investigation of the Enhancement of Functionality in Common Genes

In order to enhance comprehension of the fundamental biological roles of genes associated with CD and RA, the GO and KEGG pathways were examined using the “ClusterProfiler”, “ggnewscale”, and “DOSE” packages in the R package [16-18], a significance level of adjusted *p*< 0.05 was accepted as statistically important.

### 2.5 Identification of CD-associated Junction Genes in RA

A search tool for the retrieval of interacting genes *via* the Internet (STRING, version 12; https://cn.string-db.org/) enables the identification of interactions between enzymes of interest, including immediate binding relationships, and to construct the PPI network with intricate regulatory connections. Interactions that had a cumulative score greater than 0.3 were deemed to be statistically significant. We visualized this PPI network of hub genes using Cytoscape (version 3.7.2). The GeneCards database (http://www.gene cards.org/) was subsequently deployed to extract further details on the hub genes, including their whole name, alternative names, and biological function.

### 2.6 Determining CD and RA Co-morbidity Genes with Machine Learning

Gene screening accuracy can be enhanced by the utilization of machine learning techniques. The LASSO logistic regression method selected 24 hub genes for inclusion in the model that used multiple regression. It achieved this by increasing a penalty function and gradually shrinking the coefficients. As a result, the model was simplified and only the genes with the greatest degree of fit were retained. Using the “glmnet” package [19], LASSO regression was managed to discover hub genes.

### 2.7 Assessment of Key Gene Variant Diagnostic Accuracy

The R packages “PerformanceAnalytics” [20] and “circlize” [21] were put to conduct correlation analysis on hub genes. The “corrplot” package was employed to show the obtained data. Furthermore, the “limma” tool was implemented to validate distinguishing expression evaluation for hub gene differences. To validate the hub genes, we assessed the variability of these genes’ expression in additional datasets pertaining to RA and UC (GSE6731 and GSE55457). By comparing data from two datasets and constructing two WGCN networks from the two expression matrices, the “WGCNA” R program was leveraged. Select the module from the combined CD and RA datasets that satisfies the requirements based on the result of the calculation utilizing the online tool EVenn [22]. Furthermore, ROC analyses were performed on the GSE6731, GSE55457, GSE95095, and GSE1919 datasets to determine whether hub genes might distinguish CD and RA cases from the corresponding control samples. The research was conducted utilizing the “pROC” R package [23].

### 2.8 Analysis of Immune Infiltration

The gene lists for immunoreactions, collected from the ImmPort database (https://www.immport.org/), encompass many categories of innate and adaptive responses. The “ggplot2”, “limma”, and “pheatmap” software packages were employed to analyze the function pathways that exhibited enrichment with hub genes. A technique called ssGSEA evaluates how important genes are enriched in a specified dataset. In addition, we conducted ssGSEA using the “GSEABase” [24] and “GSVA” [25] packages to examine variations in the expression of immune cells between CD and RA samples, as well as the immune infiltration of hub genes.

## 3 RESULTS

### 3.1 Data Variance Analysis and Visualization

**Fig. (2):**
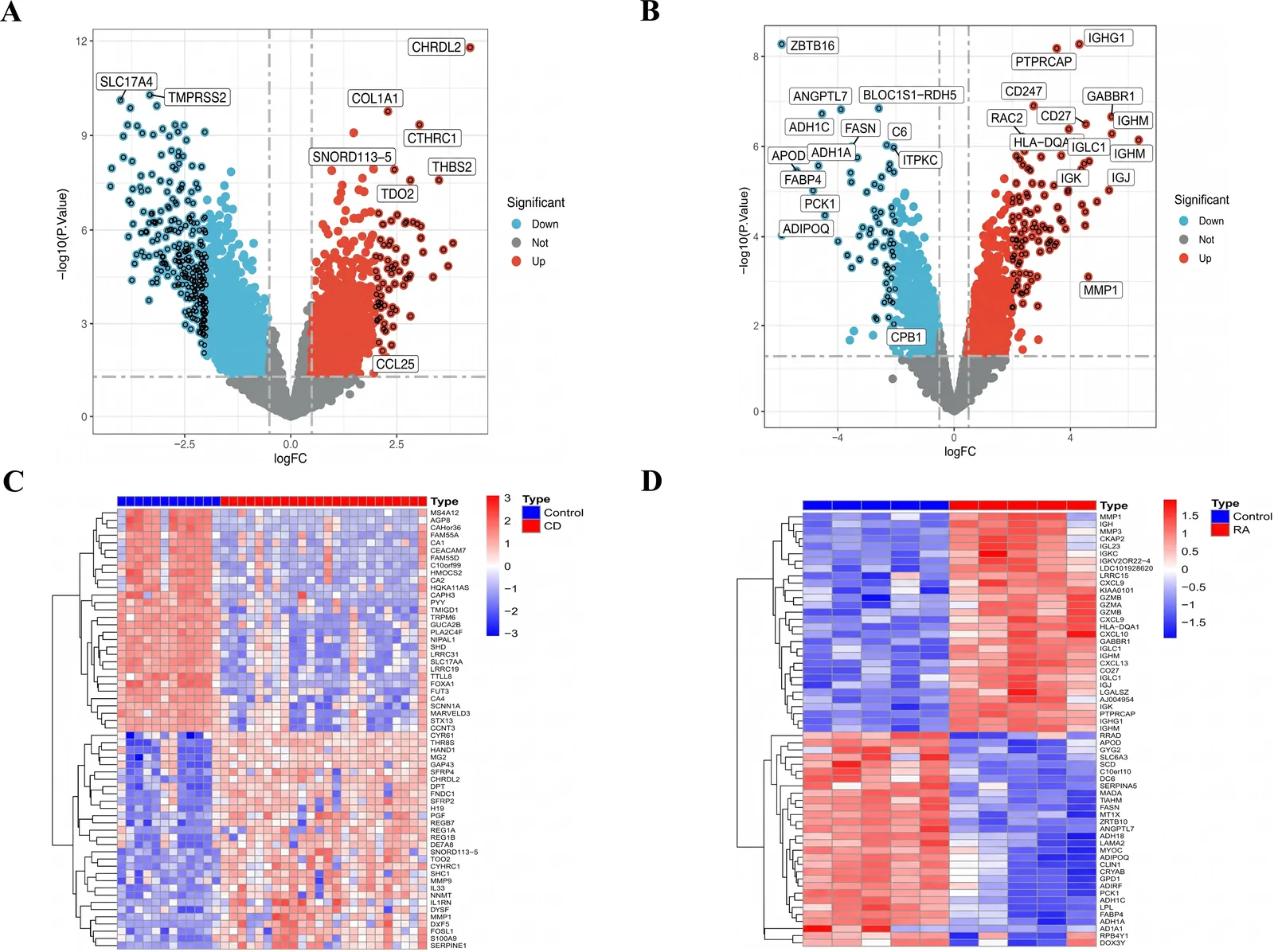
(**A, B**) The volcanoes map of GSE95095 and GSE1919. (**C, D**) The heatmaps of GSE95095 and GSE1919.

Fig. (**1**) illustrates the technical route of this study. The microarray findings were standardized, and “pheatmap” and “ggplot2” were selected to create heatmaps and volcano maps. These maps were generated based on an adjusted *p*- value of less than 0.05 and |log2FC| >1. Genes displaying significant alterations were identified through an adjusted *p*- value of less than 0.05 and |log2FC|>2.5 (Figs. **2A**-**D**). We found 473 genes with up-regulated expression and 1042 genes with down-regulated expression in CD, 221 expression was up-regulated and 199 down-regulated in RA.

### 3.2 WGCNA Identifies Key Module Genes

**Fig. (3):**
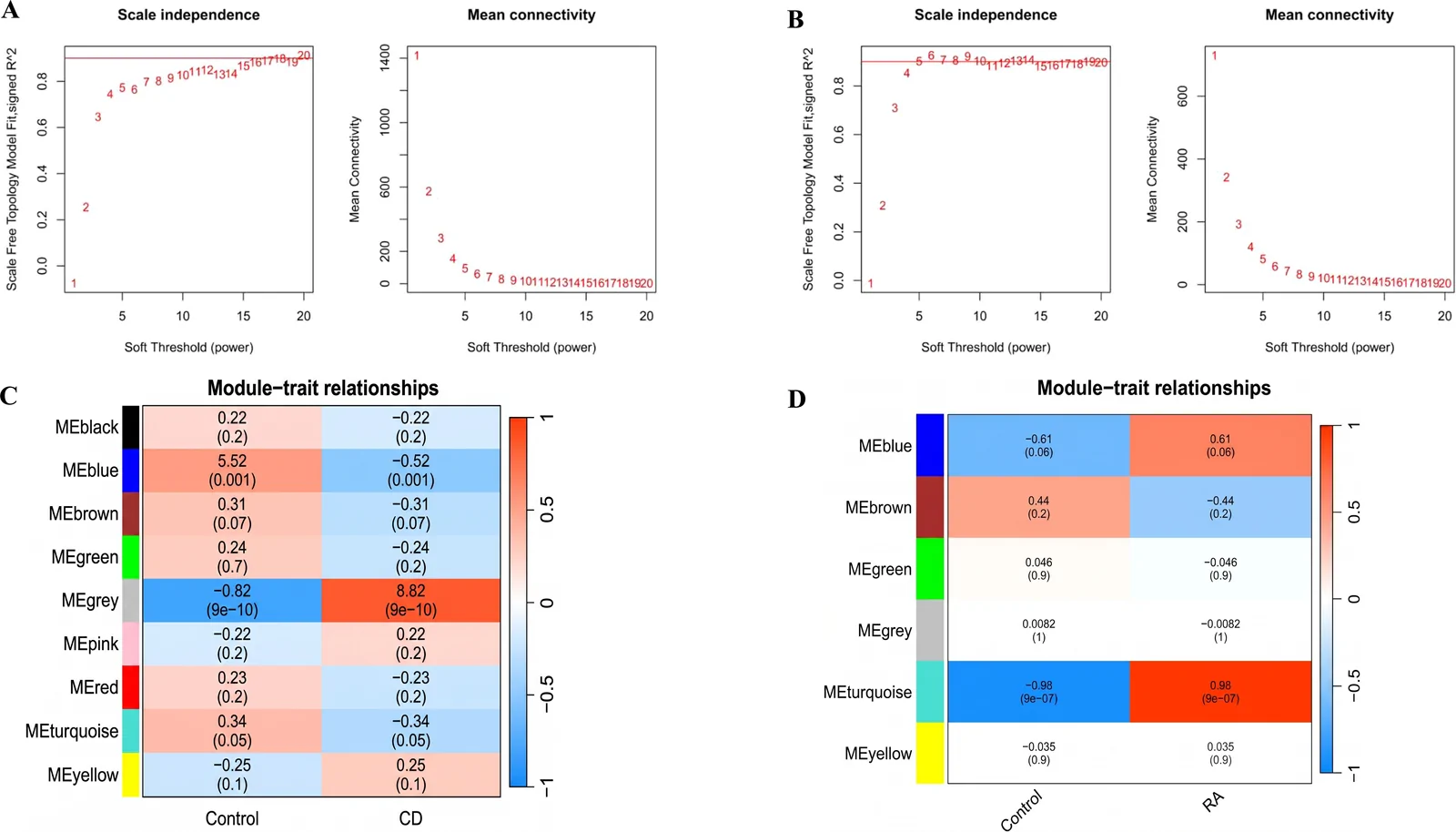
(**A, B**) Definition of soft threshold power through Weighted Gene Co-Expression Network Analysis (WGCNA). Scale-free indices and mean connectivity were analyzed for various soft threshold powers(β). (**C, D**) A gene dendrogram was created by averaging the results of a chain hierarchy clustering algorithm. The colorful rows underneath the tree diagram indicated the assignment of modules based on dynamic tree cutting.

We implemented WGCNA to identify gene modules that exhibit co-expression in the UC and RA datasets. As the sample of selected databases encompassed a range of disease types, it was necessary to exclude these data points prior to analysis. The remaining data were then divided into control and disease groups. Subsequently, the soft threshold power for GSE95095 and GSE1919 was determined to be fifteen and four, respectively, based on a scale independence larger than 0.90. This was done to ensure the creation of physiologically meaningful scale-free networks, as shown in (Figs. **3A** and **B**). We classified the genes into 9 and 6 modules, respectively, using the dynamic branching cut approach to the datasets GSE95095 and GSE1919. By utilizing GS and MM calculations, the major modules associated with CD and RA were identified, enabling the connection of these modules with clinical features. The figures depicted the correlation between clinical features in both the control and illness groups. The module eigengene MEgrey module was strongly associated with CD (Fig. **3C**), whereas the MEturquoies module was significantly associated with RA (Fig. **3D**).

### 3.3 Acquisition of Overlapped Genes and Hub Genes

**Fig. (4):**
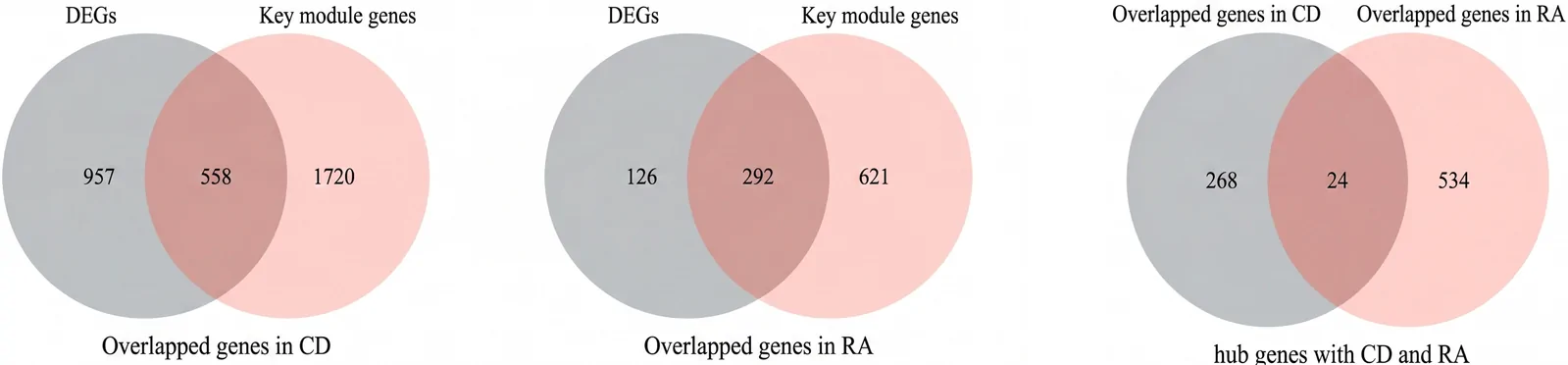
(**A, B**) Overlapped DEGs and Key module genes in CD and RA. (**C**) 24 hub genes overlepped to CD and RA.

By overlapping DEGs and MEgrey module genes in CD, we obtained 558 overlapped genes (Fig. **4A**). Similarly, we obtained 292 overlapped genes in RA (Fig. **4B**). Finally, we obtained 24 hub genes common to CD and RA (Fig. **4C**).

### 3.4 Determining the Function of Common Genes

**Fig. (5):**
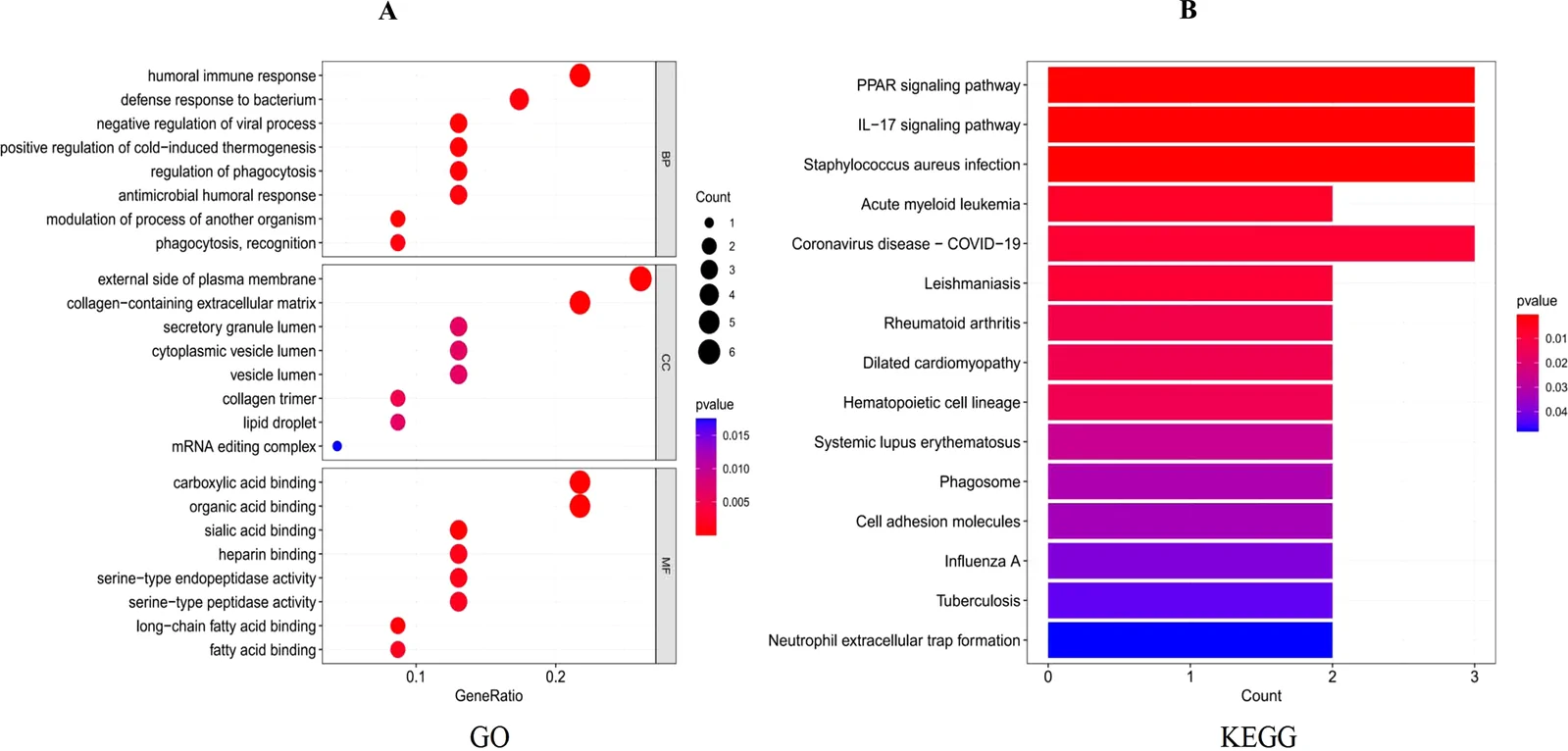
(**A, B**) Gene Ontology (GO) enrichment and Kyoto Encyclopedia of Genes and Genomes (KEGG) pathway results for the 24 hub genes. *P*< 0.05 was considered significant.

Examining the GO and KEGG pathways allowed researchers to study the functional expression variations of 24 hub genes. First, the GO analysis showed that these common genes were mostly involved in controlling phagocytosis, the production of heat in response to cold, the immune system's response to bacteria, and the humoral immune response with biological processes (BP). The extracellular matrix containing collagen and the outside of the plasma membrane were found to be positive in cellular components (CC), and heparin binding, sialic acid binding, organic acid binding, and carboxylic acid binding were all positively linked with molecular function (MF) (Fig. **5A**). Leishmaniasis, acute myeloid leukemia, staphylococcus aureus infection, the PPAR and the IL-17 signaling pathway were all clarified to have these genes in common enriched in the KEGG study (Fig. **5B**).

### 3.5 PPI Network Analysis

We applied Cytoscape software to produce a PPI network of hub genes with a total score greater than 0.3 There are 21 nodes and 40 edges in the network, the nodes represent the various genes, and the edges represent the existence of interactions between individual genes; the redder the color of a node, the stronger the connection between that gene and other genes (Supplementary material **S1**). The 10 genes with the strongest linkage strength were calculated using the ctyoHubba-DMNC algorithm in Cytoscape as FCGR1A, SELL, FCN1, GZMK, CD27, S100A9, CXCL10, ADIPOQ, MMP1, and SLPI.

### 3.6 Machine Learning Identifies Common Hub Genes

**Fig. (6):**
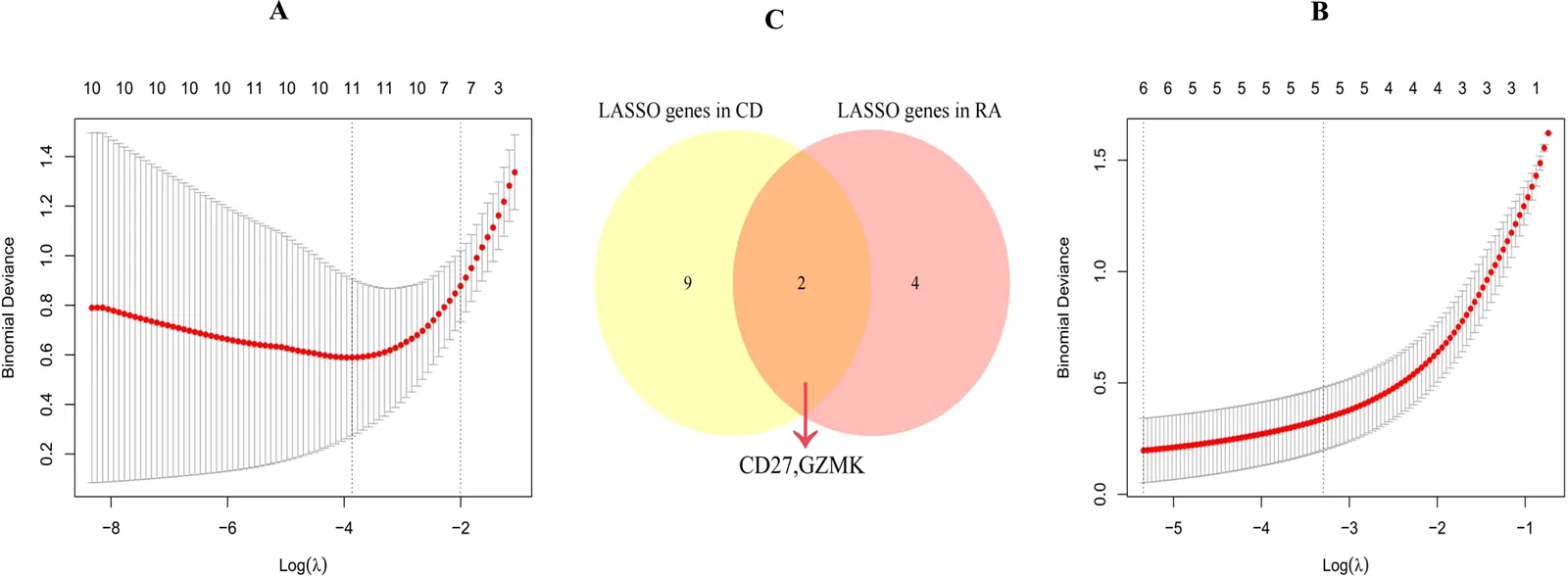
(**A, B**) LASSO logistic regression algorithm is used to retain the most predictive features and tuning parameter selection in the LASSO model. (**C**) Overlapped CD and RA machine learning to screen genes,two potential biomarker genes were obtained.

The LASSO algorithm was used to screen hub genes among 24 common genes in CD and RA. Eleven important genes in CD were found by the LASSO regression algorithm. These genes were GAP43, HLA-DOB, MMP1, FABP4, CD27, CYR61, GZMK, PIM2, SELL, SLPI, and FGFBP1 (Fig. **6A**). The LASSO algorithm found eight important genes in RA. These were FCN1, AQP9, CD27, GZMK, ADAMTS2, and FCGR1A (Fig. **6B**). LASSO identified CD27 and GZMK as common hub genes in CD and RA by overlapping their chosen genes (Fig. **6C**). Table **1** in the Gene Cards database presents the complete names and functions of the entities.

### 3.7 Verification of the Diagnostic Performance of Common Hub Genes

**Fig. (7):**
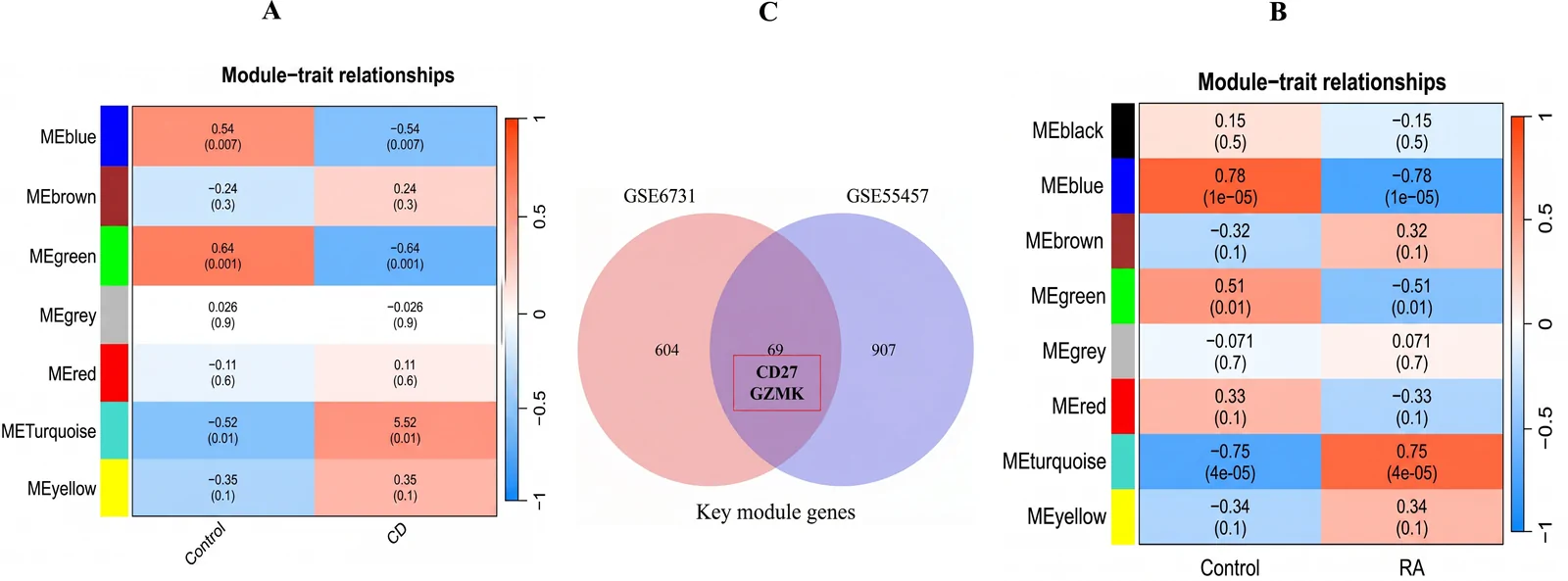
(**A, B**) Based on CD and RA validation data sets(GSE6731 and GSE55457), the gene tree is obtained by averaging the results of the chain hierarchy clustering algorithm. The colored rows below the tree represent module allocation based on dynamic tree cutting. (**C**) and overlap the key module genes.

We confirmed the hub genes by examining GSE6731 and GSE55457. Initially, we performed WGCNA to identify genetic units that exhibit co-expression patterns in both the CD and RA datasets. The eigengene module MEturquoies had a high association with CD (Fig. **7A**), and the MEturquoies module had an intense link with RA (Fig. **7B**). Then, we performed a gene overlap analysis across two modules, resulting in the identification of 69 genes that were similar to both. Among these common genes, we further identified two main hub genes (Fig. **7C**).

**Fig. (8):**
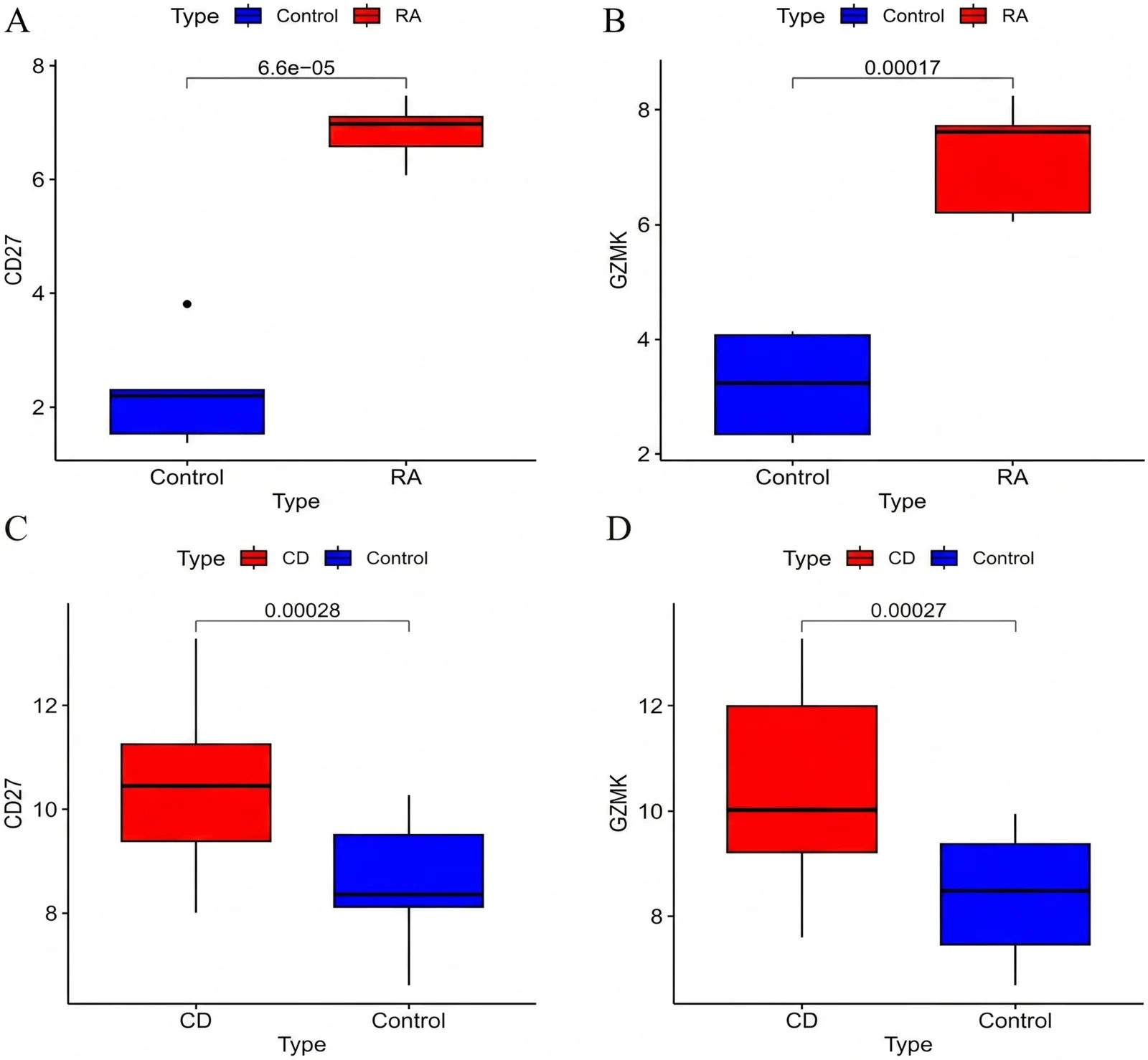
(**A-D**) In the differential expression of two potential biomarker genes in GSE95095 and GSE1919, CD27 and GZMK were highly expressed in both CD and RA, and the expression difference was statistically significant (*p*<0.001).

**Fig. (9):**
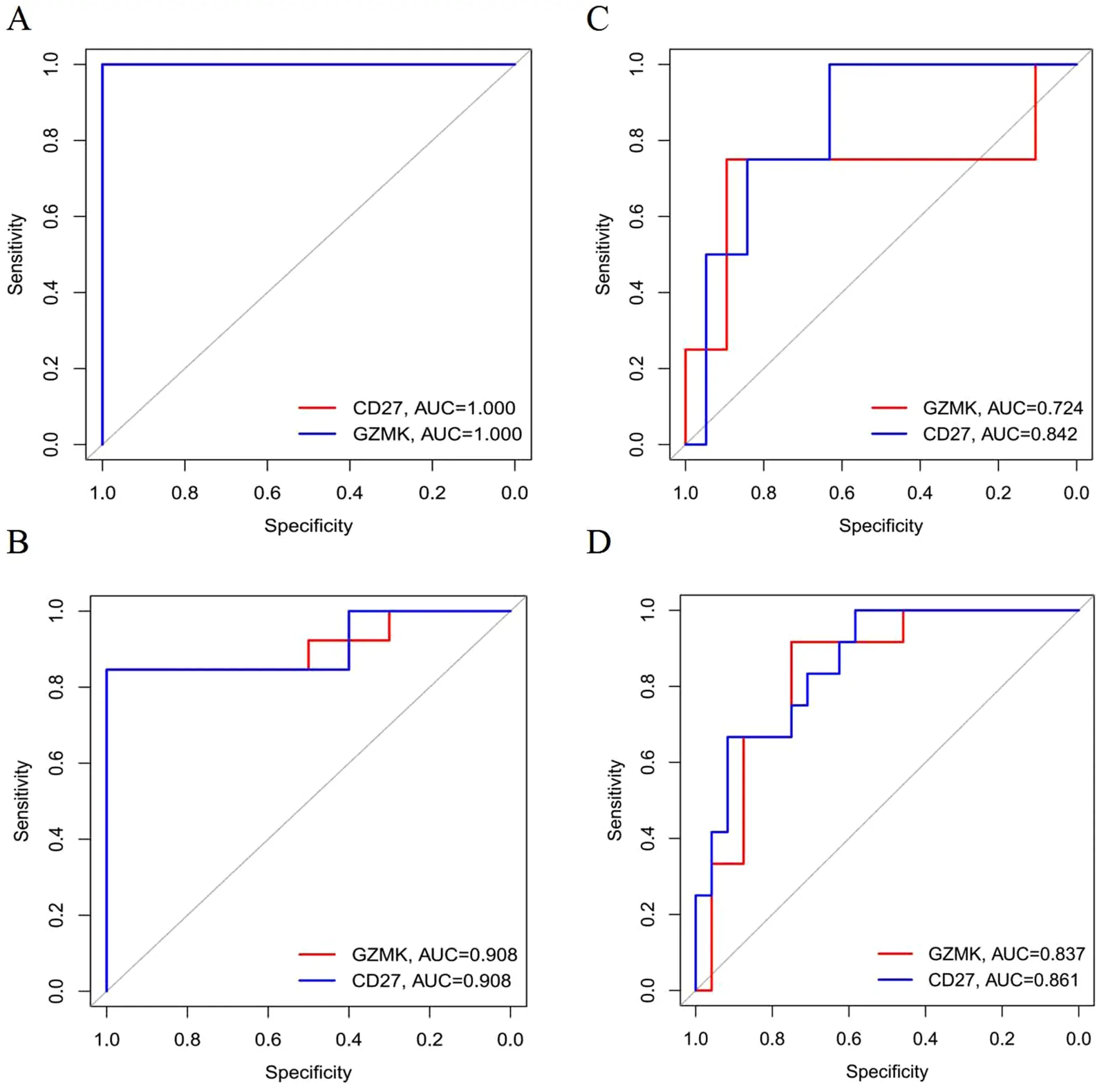
Evaluation of the diagnostic implications for potential biomarkers. (**A-D**) ROC of CD27 and GZMK in dataset GSE1919, GSE55457, GSE6731, and GSE95095.

**Fig. (10):**
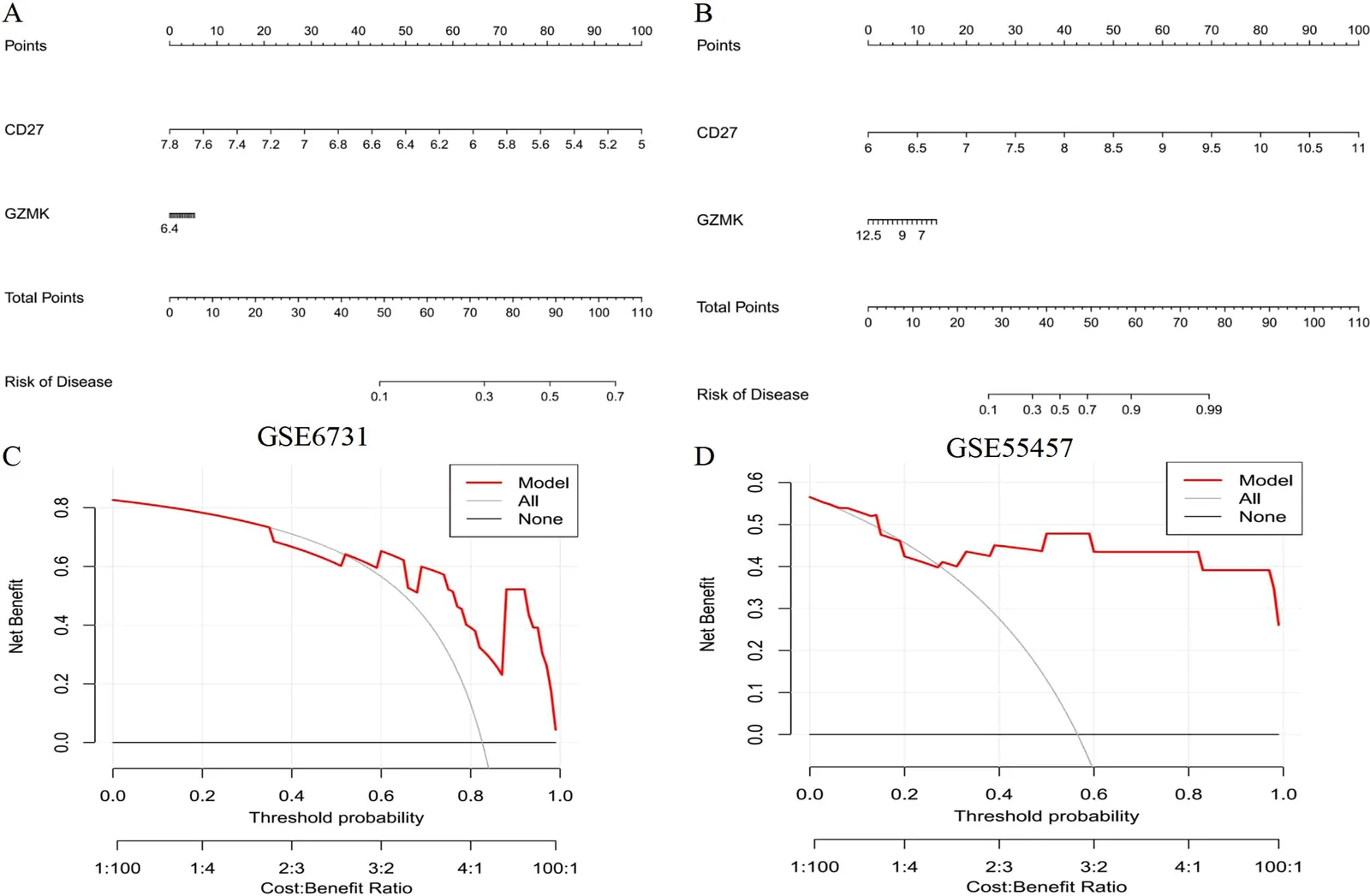
(**A, B**) A developed nomogram for the prognostic prediction of CD and RA potential biomarker genes. (**C, D**) This graph shows the predicted scores after aggregation of two hub biomarker genes proportions.

Differential expression of two hub genes in GSE95095 and GSE1919 was calculated using the “ggsci”, “ggpubr,” and “limma” R packages. We found that CD27 and GZMK were highly expressed in both CD and RA, and the difference in expression was significant (*p*<0.001) (Figs. **8A** and **D**). We therefore suggest that these two hub genes may play important roles in CD and RA. Using the “pROC” R package, this research compared the two core genes’ diagnostic performance across four datasets. The results were as follows: the AUC both of CD27 and GZMK in GSE1919 was 1 (Fig. **9A**); the AUC of CD27 and GZMK in GSE6731 was 0.724 and 0.842 (Fig. **9B**); the AUC both of CD27 and GZMK in GSE55457 was 0.908 (Fig. **9C**); and the AUC of CD27 and GZMK in GSE95095 was 0.873 and 0.861 (Fig. **9D**). The model that included CD27 and GZMK was the result of using a nomogram to assess the diagnostic significance of two hub genes (Figs. **10A** and **B**). We also constructed the calibration curve and decision curve using the “ggDCA”, “rms, nricens, foreign, rmda, andregplot” R packages (Figs. **10C** and **D**). After observing the prediction model of CD27+GZMK we found that the contribution of GZMK in the model was small. In order to make our model easier for future research and clinical work, we analyzed the prediction model of the CD27 single gene and found that the CD27 single gene model has similar prediction ability (Supplementary material **S2**). This displayed a strong correlation between two hub genes for CD and RA.

### 3.8 Analysis of Immune Infiltrating Cells

**Fig. (11):**
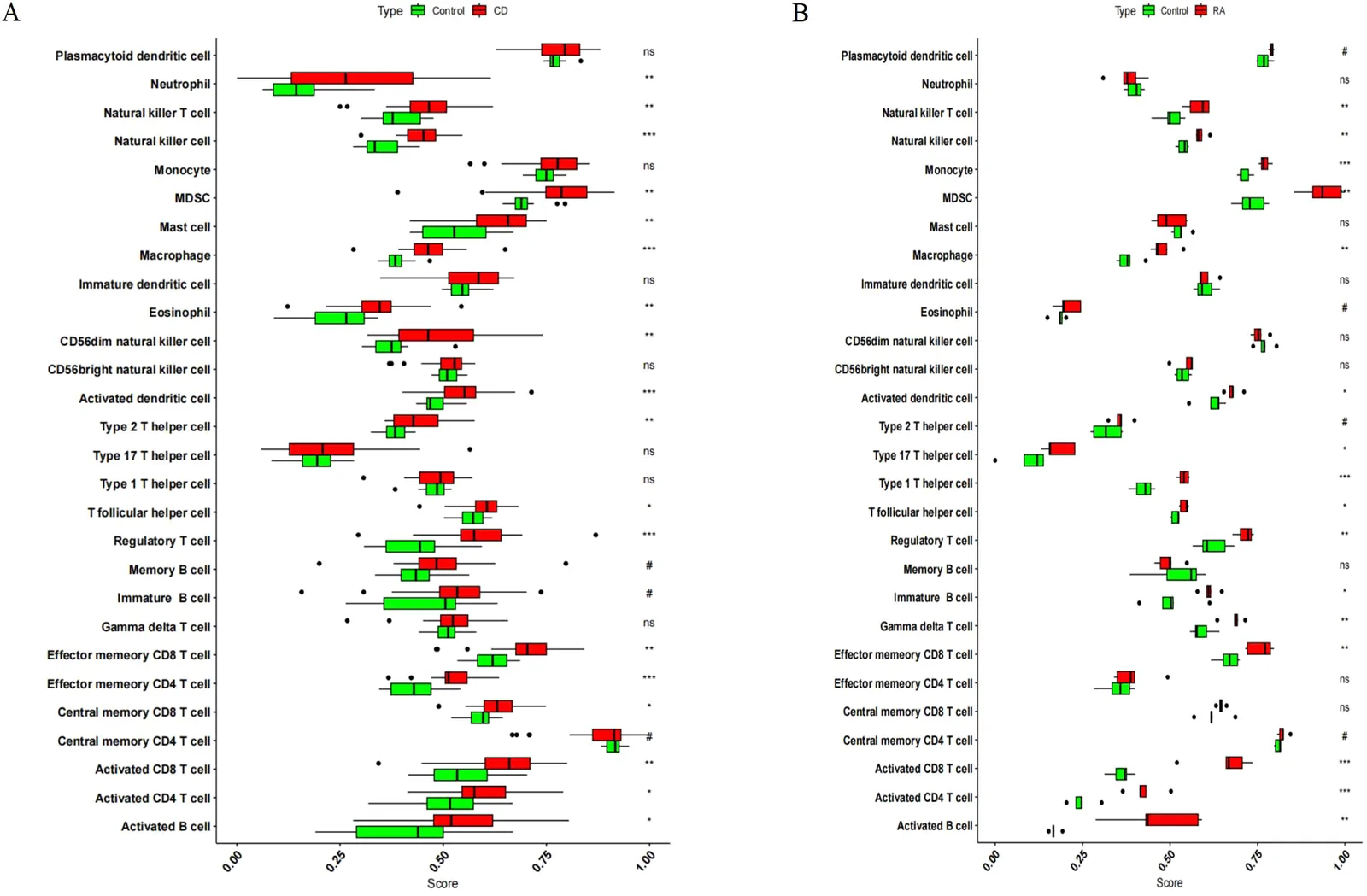
(**A, B**) Expression differences of 28 immune infiltrating cells in samples of CD and RA. (^ns^*P* < 1, ^#^*p* < 0.2, *p < 0.05, **p < 0.01, ***p < 0.001).

A correlation analysis was conducted between 24 hub genes and 28 distinct types of immune-infiltrating cells. Initially, we evaluated the differences in expression between 28 immune infiltrating cells in the GSE95095 and GSE1919 datasets. The table of 28 immune cell types in GSE95095 showed that the natural killer cell, macrophage, regulatory T cell, and effector memory CD4 T cell were substantially in line with CD (*p*<0.001). CD was strongly connected with neutrophil cells, natural killer T cells, MDSC, mast cells, eosinophils, CD56dim natural killer cells, type 2 T helper cells, effector memory CD8 T cells, and activated CD8 T cells (*p*<0.01). Activated B cell helper cells, activated CD4 T cells, central memory CD8 T cells, and T follicular helper cells were positively associated with CD (*p*<0.05) (Fig. **11A**). In GSE1919, it was indicated that monocytes, MDSC, type 1 T helper cells, activated CD8 T cells, and activated CD4 T cells had an enormously beneficial connection with CD (*p*<0.001). RA had an elevated correlation with the activation of B cells, gamma delta T cells, regulatory T cells, macrophages, natural killer cells, and natural killer T cells (*p*<0.01). Immature B cells, T follicular helper cells, Type 17 T helper cells, and activated dendritic cells were positively associated with RA (*p*<0.05) (Fig. **11B**).

### 3.9 Enrichment Analysis of CD27 and GZMK in CD and RA

**Fig. (12):**
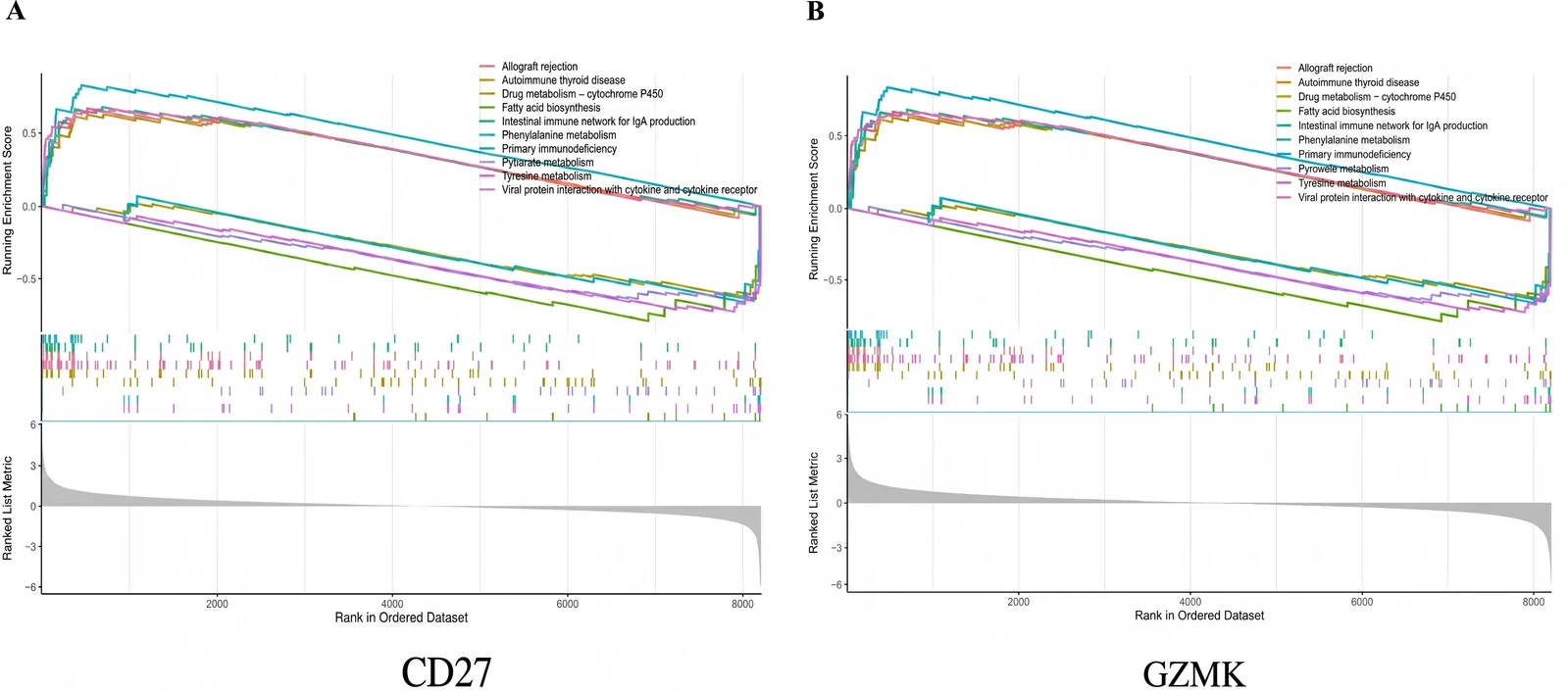
Single gene GSEA analysis. (**A, B**) The 5 most significant up_GSEA and down_GSEA results for CD27 and GZMK in the RA dataset.

**Fig. (13):**
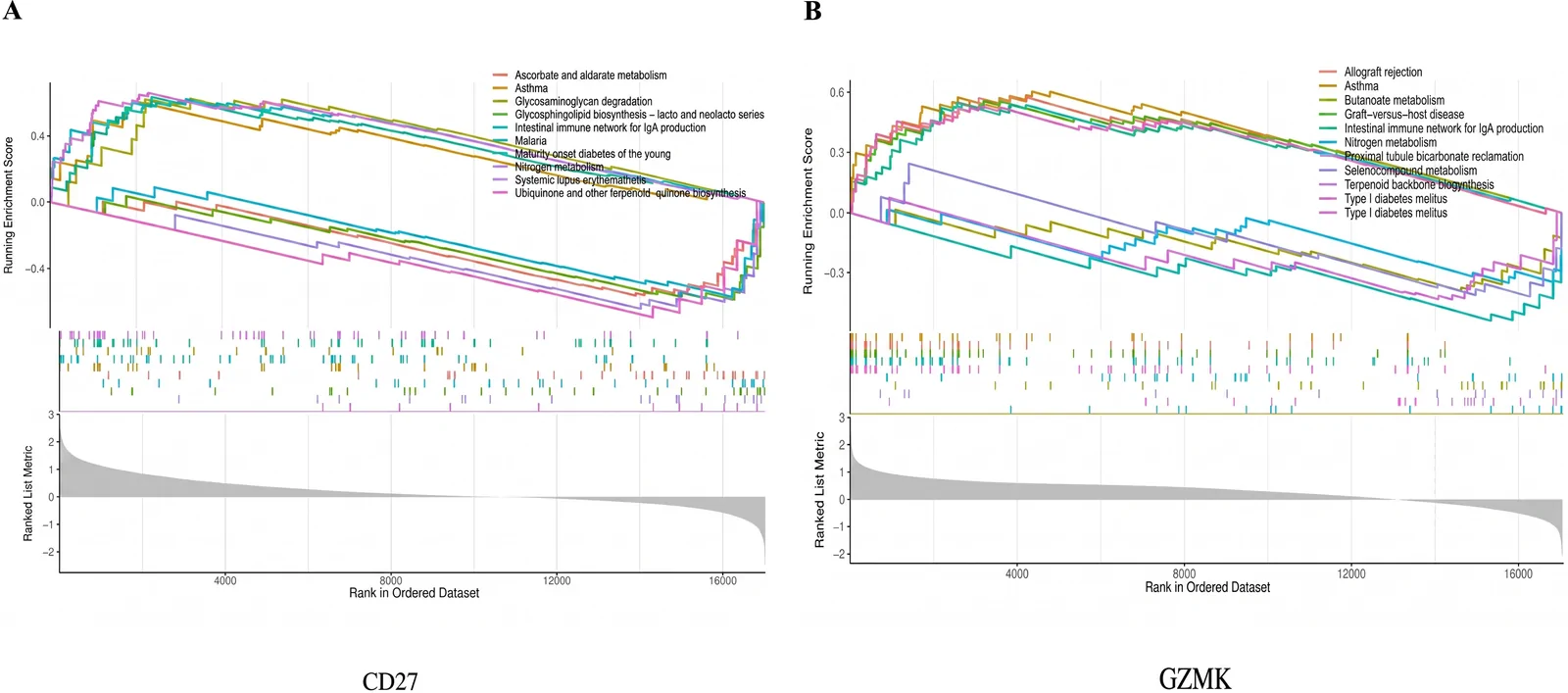
Single gene GSEA analysis. (**A, B**) The 5 most significant up_GSEA down_GSEA results for CD27 and GZMK in the CD dataset.

With the goal to investigate the pathways governed by CD27 and GZMK, a single-gene GSEA analysis was conducted, yielding the subsequent findings: CD27 and GZMK in patient CD positively affect ascorbate and aldarate metabolism, asthma, the breakdown of glycosaminoglycans, the biosynthesis of glycosphingolipids, the lacto and neolacto series, and the gut immune system makes IgA. CD27 and GZMK mainly negatively affected malaria, young-onset diabetes, nitrogen metabolism, systemic lupus erythematosus, ubiquinone, and other terpenoid-quinone biosynthesis in patient CD (Figs. **12A** and **B**). CD27 and GZMK in patient RA mainly affected allograft rejection, autoimmune thyroid disease, drug metabolism-cytochrome P450, fatty acid biosynthesis, and the intestinal immune network for IgA production. CD27 and GZMK are mainly negatively affected. Phenylalanine metabolism, primary immunodeficiency, pyruvate metabolism, tyrosine metabolism, and viral protein interaction with cytokine and cytokine receptor (Figs. **13A** and **B**). Notably, the results of the TOP 5 upGSEA and TOP 5 downGSEA enrichment analysis of CD27 and GZMK in RA were consistent, which may indicate the similarity of their pathogenic mechanisms in RA. In addition to this, we analyzed the relation between CD27, GZMK and 28 immune cells, and the findings have shown that CD27 and GZMK were closely associated with many kinds of immune cells both in CD and RA, the results showed that CD27 and GZMK were closely associated with NK cells, CD8 T cells, B cells, etc. both in RA and CD (Supplementary material **S3**).

## 4 DISCUSSION

In this study, we identified co-morbidity genes for CD and RA using machine learning and bioinformatics approaches. Among the candidate genes, CD27 and GZMK demonstrated superior diagnostic efficacy across four independent datasets, highlighting their potential as biomarkers for both diseases. CD27 is a protein-coding gene belonging to the TNF receptor superfamily, which plays a critical role in the generation and maintenance of T-cell immunity [26]. Mutations in CD27 have been linked to combined immunodeficiency [27] and lymphoproliferative syndrome 2 [28]. Functionally, CD27 is involved in key signaling pathways, including the Akt pathway and TNF superfamily interactions, which regulate immune responses and apoptosis [29, 30]. Additionally, CD27 participates in transmembrane signaling and modulates cysteine-type endopeptidase activity, enabling cells to respond to external stimuli [31, 32].

GZMK, another hub gene identified in our study, encodes a serine protease found in the cytoplasmic granules of cytotoxic lymphocytes [33]. It is primarily associated with the granzyme pathway, which mediates serine-type endopeptidase and peptidase activities [34, 35]. GZMK has been implicated in various diseases, including malignant tumors, suggesting its broader role in immune dysregulation [36].

Immune dysregulation is a hallmark of both RA and CD, contributing significantly to their pathogenesis [1, 4]. In our study, we observed elevated expression of CD27 and GZMK in both diseases, underscoring their potential as biomarkers. CD27, a well-established immune-related gene, plays a pivotal role in T-cell activation and immune regulation. The CD27-CD70 interaction enhances CD4^+^ T lymphocyte proliferation and proinflammatory cytokine production while blocking this pathway has been shown to ameliorate RA in animal models [37]. This interaction also impairs the function of regulatory B10 cells, further exacerbating RA pathology [38].

In RA patients, CD27 is frequently overexpressed in immune cells, including IgD-CD27- memory B cells and CD70(+) CD4 T cells, which are associated with disease severity [39-41]. These cells contribute to RA pathogenesis by producing inflammatory cytokines such as IFN-γ and IL-17 [42, 43]. Similarly, in CD, CD27-expressing cells are enriched at intestinal lesion sites, suggesting their involvement in local immune responses [44, 45, 47]. Notably, CD27 has been identified as a predictive marker for TNF therapy efficacy in CD, highlighting its clinical relevance [46]. Furthermore, reduced numbers of CD24^hi^CD27^+^ B cells and decreased IL-10 production have been observed in both RA and CD, indicating a shared mechanism of immune dysregulation [48]. These findings collectively demonstrate the critical role of CD27 in the pathogenesis of RA and CD, supporting its potential as a reliable biomarker.

GZMK, another key gene identified in our study, is highly expressed in CD8^+^ T cells and has been implicated in the pathogenesis of both RA and CD. In RA, GZMK^+^CD8^+^ T cells are enriched in synovial tissue, where they contribute to synovitis and joint destruction by targeting citrullinated antigens [49, 50]. Single-cell sequencing studies have further revealed that GZMK^+^ CD8^+^ T cells exhibit a high degree of clonality in RA synovium, suggesting their role in disease progression [51]. Notably, GZMK-expressing CD8^+^ T cells are also found in the gut of CD patients, indicating a cross-disease immune population that may link RA and CD [52, 53]. In CD, GZMK+ cells in the lamina propria are associated with inflammation, further supporting its role in immune dysregulation [54]. Additionally, GZMK may modulate immune responses through the endoplasmic reticulum stress pathway, providing a potential mechanistic link between immune activation and disease pathology [35, 55]. These findings highlight the dual role of GZMK in RA and CD, suggesting its potential as a therapeutic target.

Both innate and adaptive immunity play critical roles in the pathogenesis and progression of RA and CD [56-58]. Through ssGSEA immune infiltration analysis, we identified a close association between various immune cell populations and the development of both diseases. In CD, the innate immune response in the intestine is mediated b y neutrophils, monocytes, macrophages, and dendritic cells (DCs), as well as innate lymphoid cells (ILCs) and natural killer (NK) cells, which provide rapid and non-specific immune responses [56, 59, 60]. Dendritic cells in CD patients play a pivotal role in regulating immune tolerance and activation by modulating effector T cells, regulatory T cells, Th17 cells, and monocyte-macrophage subsets [60, 61]. Additionally, the Mincle/Syk pathway in macrophages promotes chemokine production and neutrophil recruitment *via* MAPK activation, contributing to intestinal inflammation [62, 63]. This pathway may also intersect with GZMK-related signaling, further linking immune dysregulation to disease pathology [35].

CD4^+^ T cells, particularly the CD-specific CD4^+^ Trm subset, are major producers of type 1 inflammatory cytokines in response to various stimuli, highlighting their role in CD pathogenesis [64]. Mast cells, while essential for maintaining barrier defenses, can also exacerbate inflammation in the intestinal tract, as seen in CD [65]. In dendritic cells, muramyl-dipeptides synergize with LPS to enhance inflammatory cytokine production and co-stimulatory molecule expression, promoting the proliferation of Th2-like CD4^+^ T cells and driving pro-inflammatory responses in CD [66]. Furthermore, mucosal CD103-CD8^+^ T cells in CD patients exhibit altered transcriptomes, including upregulation of Th17-related genes such as CCL20, IL22, and IL26, which may further contribute to disease progression [67].

In RA, synovial tissue macrophage counts are a reliable indicator of disease severity and treatment response [68, 69]. Conversely, a reduction in circulating DCs is associated with heightened inflammation [70], while NK cells accumulate in inflammatory lesions and interact with CD14^+^ monocytes to amplify inflammation and promote bone destruction [71]. Naïve CD4^+^ T cells, upon activation by antigen-presenting cells (APCs), differentiate into various subsets, including Th17 cells, which drive autoimmune inflammation and synovitis in RA [72-74]. Imbalances in CD4^+^ T cell subsets, particularly Th17 cells, disrupt immune homeostasis and contribute to RA pathogenesis by promoting excessive cytokine production and autoantibody generation [72, 75].

The consistency between our findings and existing literature underscores the reproducibility and reliability of our study. Importantly, our analysis was based on biopsy tissues from disease sites, which are considered the gold standard for clinical diagnosis. This enhances the applicability and translational value of our model for future clinical practice and research.

In summary, our study identified shared biomarkers for RA and CD, elucidated their common immune mechanisms, and established a diagnostic model with potential clinical applications. These findings provide new targets for the diagnosis and treatment of both diseases and offer a theoretical foundation for future research. However, our study has several limitations. Although we analyzed high-quality published datasets, individual differences and confounding factors in the original studies may have influenced our results. While machine learning and bioinformatics approaches yielded robust predictive power, external experimental validation is needed to further confirm our findings. In future studies, we plan to validate these results through clinical or animal experiments to strengthen the reliability and translational potential of our conclusions.

## CONCLUSION

This study is the first to combine machine learning, WGCNA, and bioinformatics exploration to analyze the genetic concordance between RA and CD, identifying CD27 and GZMK as their common diagnostic and therapeutic targets. In addition, immune cell infiltration and ssGSEA analysis suggest that the pathogenesis of CD and RA is closely related to innate immune imbalance and dysregulation of the acquired immune response system.

## Figures and Tables

**Fig. (1):**
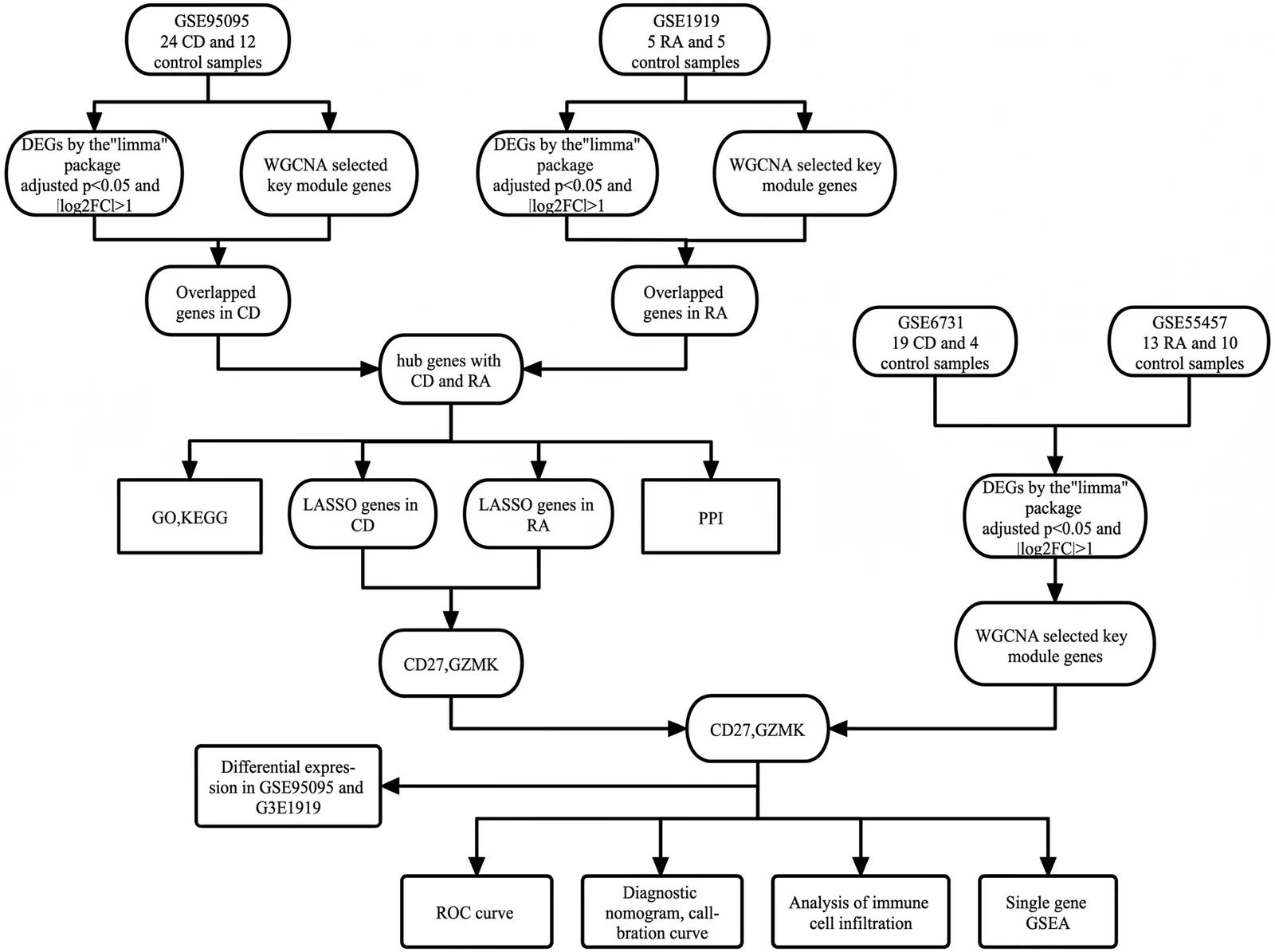
Research design flow chart.

**Table 1:** The two potential biomarker genes’ full names, alias and main function.

Gene Symbol	Full Name	Aliases	Function
CD27	CD27 Molecule	CD27 Antigen ; TNFRSF7 ; Tumor Necrosis Factor Receptor Superfamily, Member 7; T-Cell Activation Antigen CD27. TRYP2;	A Protein Coding gene. Diseases associated with CD27 include Lymphoprol iferative Syndrome 2 and Combined Immunodeficiency. Among its related pathways are Akt Signaling and TNF Superfamily - Human Ligand-Receptor Interactions and their Associated Functions. Participate in transmembrane signaling receptor activity and cysteine-type endopeptidase inhibitor activity involved in apoptotic process
GZMK	Granzyme K	NK-Tryptase-2; Fragmentin-3; PRSS; EC 3.4.21.	The product is a member of a group of related serine proteases from the cytoplasmic granules of cytotoxic lymphocytes.Among its related pathways are Granzyme Pathway.Participate in serine-type endopeptidase activity and serine-type peptidase activity

## Data Availability

The original contributions presented in the study are included in the article or Supplementary Material, further inquiries can be directed to the corresponding authors.

## LIST OF ABBREVIATIONS

RA: Rheumatoid Arthritis
CD: Crohn’s Disease
GEO: Gene Expression Omnibus
DEGs: Differentially Expressed Genes
WGCNA: Weighted Gene Co-Expression Network Analysis
GO: Gene Ontology
KEGG: Kyoto Encyclopedia of Genes and Genomes
LASSO: Least Absolute Shrinkage And Selection Operator
TOM: Topological Overlap Matrix
GS: Gene Significance
MM: Module Membership
BP: Biological Processes
CC: Cellular Components
MF: Molecular Function
DCs: Dendritic Cells
NK: Natural Killer
MAPK: Mitogen-Activated Protein Kinase
