# Supplementary material for: CD27 and GZMK as Co-Biomarkers in Rheumatoid Arthritis and Crohn's Disease and Mediate Immune Imbalance
Source: Endocr Metab Immune Disord Drug Targets. 2025 Apr 29;26:E18715303372365. doi: 10.2174/0118715303372365250421114853 (PMC13563492; doi:10.2174/0118715303372365250421114853)
Supplement: Supplementary file 1 [file EMIDDT-26-E18715303372365_SD1.pdf]

Supplementary Material

CD27 and GZMK as Co-Biomarkers in Rheumatoid Arthritis and Crohn's disease and Mediate Immune Imbalance

Li Zheng[1,2], Yong Zeng[1,*], Miao Fang[1,*], Hongquan Heng[3], Ying Luo[2] and Jie Zhong[2]

[1]Department of Orthopedics, Chengdu Second People's Hospital, Chengdu, China; [2]Department of Clinical Medicine, Chengdu Medical College, Chengdu, China; [3]Center for Joint Surgery, The First Affiliated Hospital of Army Medical University, Chongqing, China

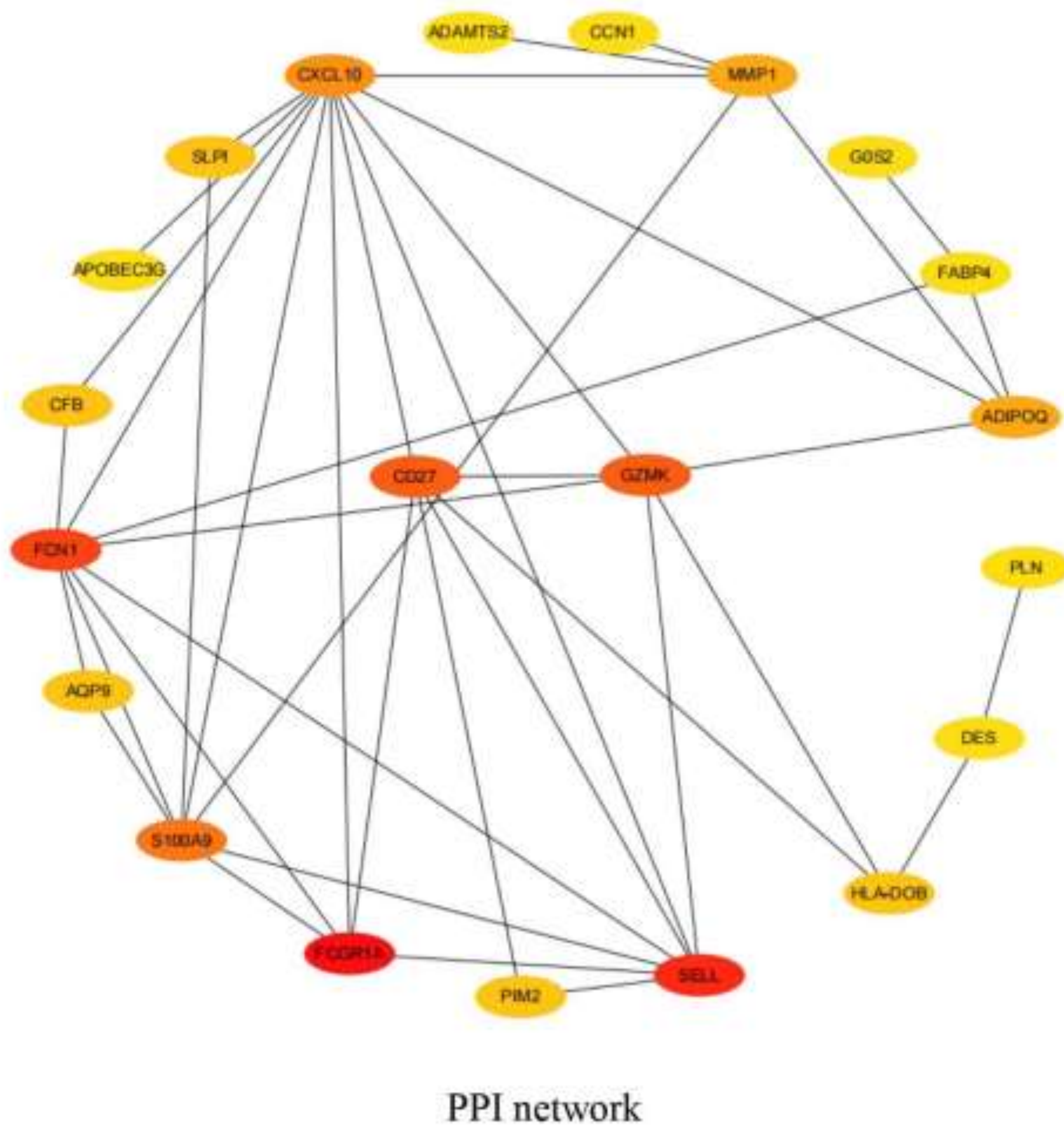

Supplementary material S1. 24 hub genes based on PPI analysis and cytoscape visualization.

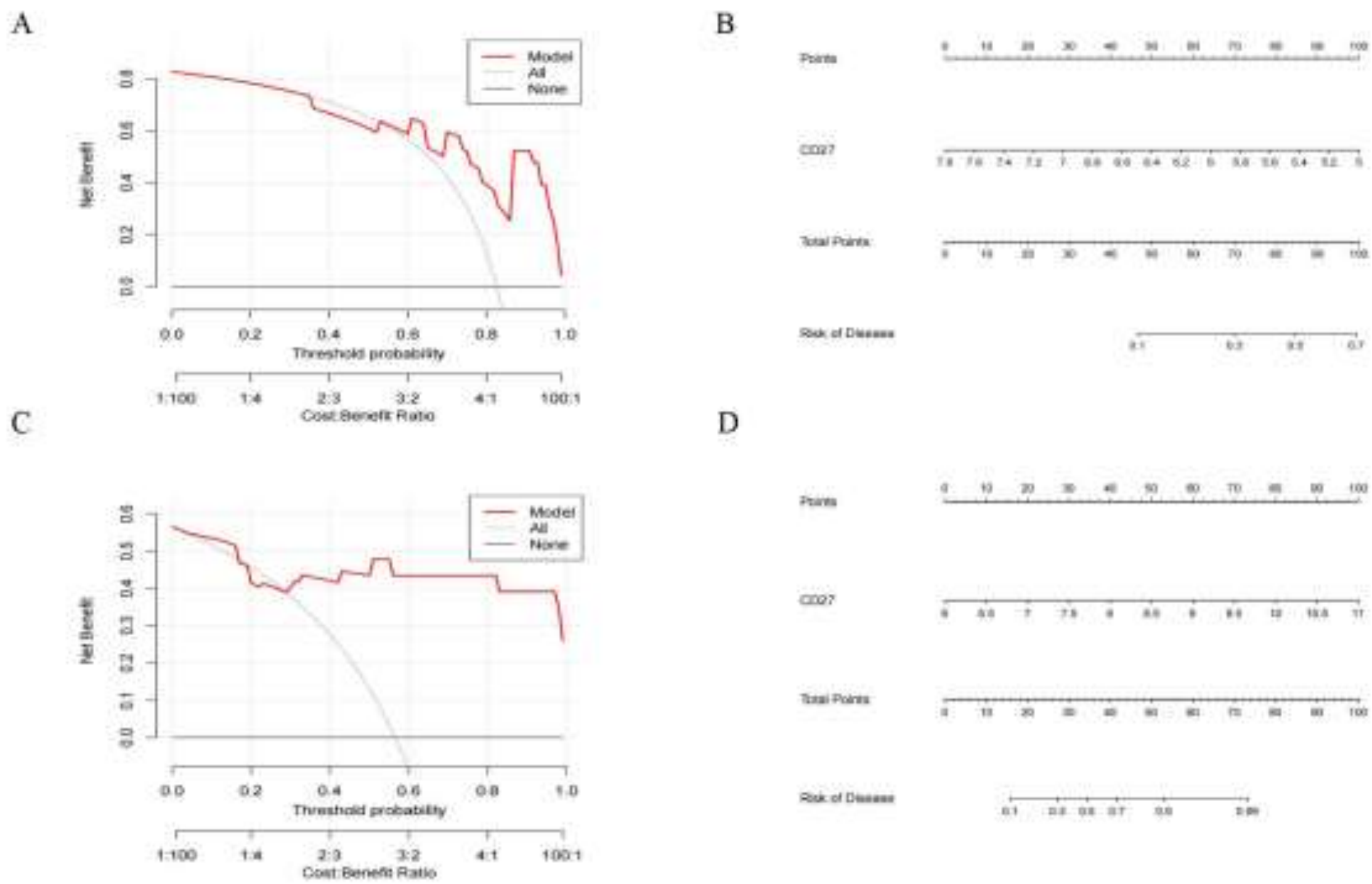

Supplementary material S2. (A) A model developed for prognostic prediction of the CD27 single gene. (B) The prediction score of this model.

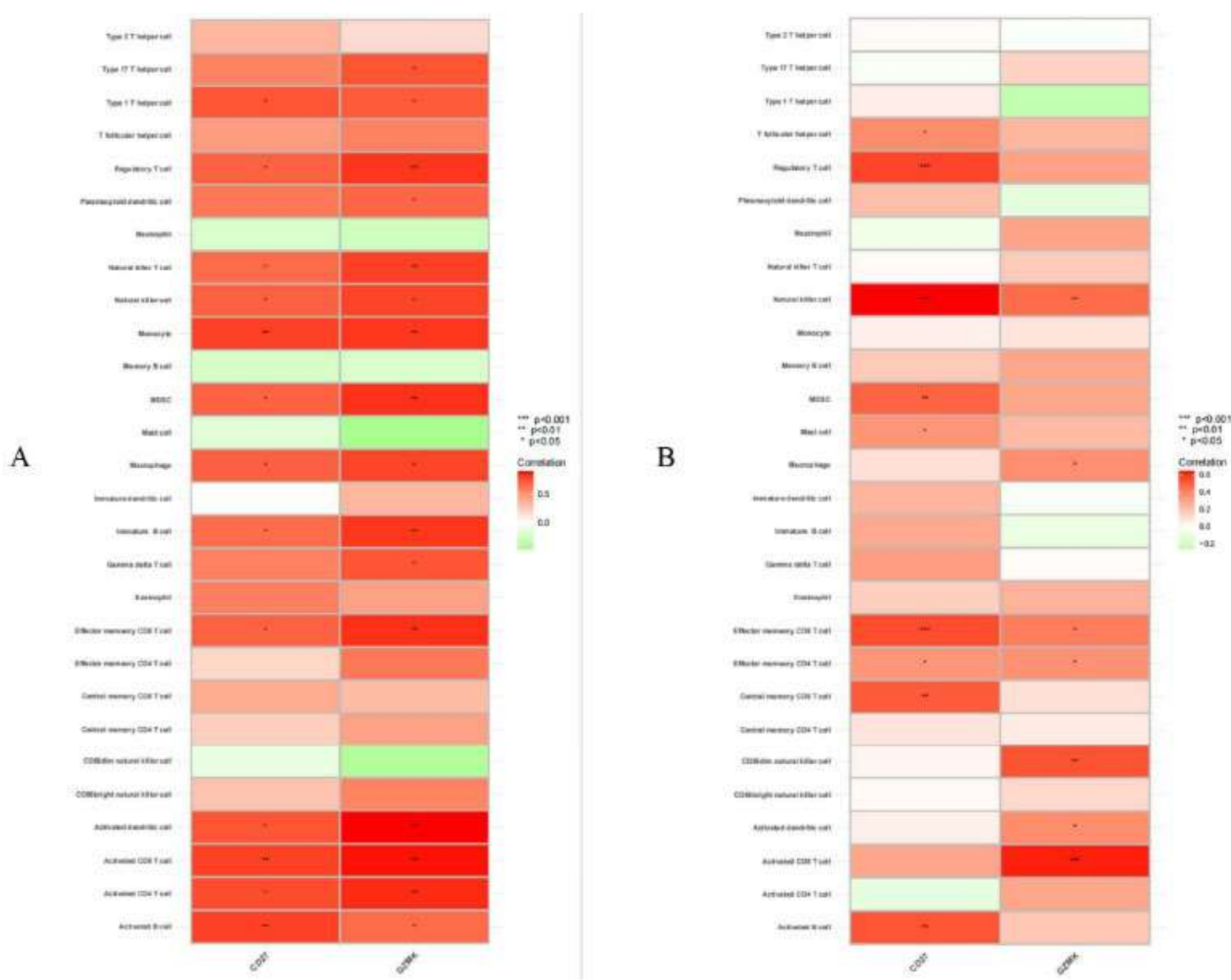

Supplementary material S3. (A,B) Correlation between CD27, GZMK and 28 immune cells in CD and RA.
